# Bromelain Extract Exerts Antiarthritic Effects via Chondroprotection and the Suppression of TNF-α–Induced NF-κB and MAPK Signaling

**DOI:** 10.3390/plants10112273

**Published:** 2021-10-23

**Authors:** Peraphan Pothacharoen, Rujirek Chaiwongsa, Theerawut Chanmee, Orapin Insuan, Thanchanok Wongwichai, Phornpimon Janchai, Pilanee Vaithanomsat

**Affiliations:** 1 Thailand Excellence Center for Tissue Engineering and Stem Cells, Department of Biochemistry, Faculty of Medicine, Chiang Mai University, Chiang Mai 50200, Thailand; peraphan.p@cmu.ac.th (P.P.); tunchanok.wong@gmail.com (T.W.); 2 Department of Medical Technology, Faculty of Associated Medical Sciences, Chiang Mai University, Chiang Mai 50200, Thailand; rujirek.c@cmu.ac.th; 3 Department of Clinical Chemistry, Faculty of Medical Technology, Mahidol University, Nakhon Pathom 73170, Thailand; theerawut.cha@mahidol.ac.th; 4 Department of Medical Technology, School of Allied Health Sciences, University of Phayao, Phayao 56000, Thailand; orapin.th@up.ac.th; 5 Unit of Excellence in Integrative Molecular Biomedicine, School of Allied Health Sciences, University of Phayao, Phayao 56000, Thailand; 6 Nanotechnology and Biotechnology Research Division, Kasetsart Agricultural and Agro-Industrial Product Improvement Institute (KAPI), Kasetsart University, Bangkok 10900, Thailand; aappmj@ku.ac.th; 7 Center for Advanced Studies in Tropical Natural Resources, National Research University-Kasetsart University, Kasetsart University, Bangkok 10900, Thailand

**Keywords:** anti-arthritis, anti-inflammation, bromelain, chondroprotection, osteoarthritis, rheumatoid arthritis

## Abstract

Bromelain, a mixture of proteases in pineapple rhizome, has beneficial biological properties. Following absorption, the compound remains biologically active in mammalian blood and tissues. Bromelain has multiple clinical and therapeutic applications because of its anti-arthritic activities. Anti-inflammation is one of the putative therapeutic effects of bromelain on osteoarthritis (OA) and rheumatoid arthritis (RA), but the molecular mechanisms in cartilage and synovial fibroblast has not been reported. Thus, in this study, interleukin (IL)-1β/oncostatin M-induced porcine cartilage and TNF-α–induced synovial fibroblast were used as the inflamed OA and RA models, respectively. The results demonstrated the chondroprotective effects of bromelain on cartilage degradation and the downregulation of inflammatory cytokine (tumor necrosis factor (TNF)-α, IL-1β, IL-6, IL-8) expression in TNF-α–induced synovial fibroblasts by suppressing NF-κB and MAPK signaling. The evidence from this study supported and explained the anti-inflammatory and analgesic effects of bromelain on arthritis in animal models and clinical studies.

## 1. Introduction

Among musculoskeletal disorders, osteoarthritis (OA) and rheumatoid arthritis (RA) are the most common prevalent disorders of the articular joints, and they cause disability and increase medical costs in elderly people. Proinflammatory cytokine-induced inflammation is the hallmark of both OA and RA.

OA is a whole-joint disease resulting in alteration of the structure of hyaline cartilage, subchondral bone, ligaments, capsules, synovial tissues, and periarticular muscles. Among proinflammatory cytokines, interleukin (IL)-1β participates in the pathogenesis of OA by upregulating matrix-degrading degraded enzymes (matrix metalloproteinases (MMPs), ADAMTS) and suppressing matrix molecules synthesis [1,2]. RA is typified by chronic inflammation of the lining of the joints with synovial inflammation and hyperplasia, hyaline cartilage degradation, bone destruction, and systemic features, including cardiovascular, pulmonary, psychological, and skeletal disorders. Tumor necrosis factor (TNF)-α is a proinflammatory cytokine plays a pivotal role in regulating the inflammatory response in RA [3].

Bromelain (EC 3.4.22.32), a proteolytic enzyme, was isolated from pineapple (*Ananas comosus*) rhizome. It has been recognized as a safe therapeutic phytochemical that exhibits clinical applications including fibrinolytic, antiedematous, antithrombotic, and anti-inflammatory effects [4,5]. The anti-inflammatory effects of bromelain were previously reported in pathological condition and cell types, such as the reduction of IL-1β, IL-6, and TNF-α secretion in immune cells [6] and downregulation of TNF-α receptor in a rat colitis model [7]. Meanwhile, IL-1β and TNF-α are the targets for OA and RA treatment, respectively. Bromelain was previously examined in a series of case reports of moderate OA and RA [8]. In OA, the pain and inflammation were reduced by oral bromelain, trypsin, and rutin administration in patients with OA [9].

Nevertheless, a molecular investigation of the anti-inflammatory effects of bromelain on chondrocytes and synovial fibroblasts is needed. Thus, in this study, the chondroprotective and anti-inflammatory effects of bromelain extract were investigated in a porcine cartilage explant model and synovial sarcoma cell line (SW982), respectively.

## 2. Results

### 2.1. HPLC Profile

**Figure 1:**
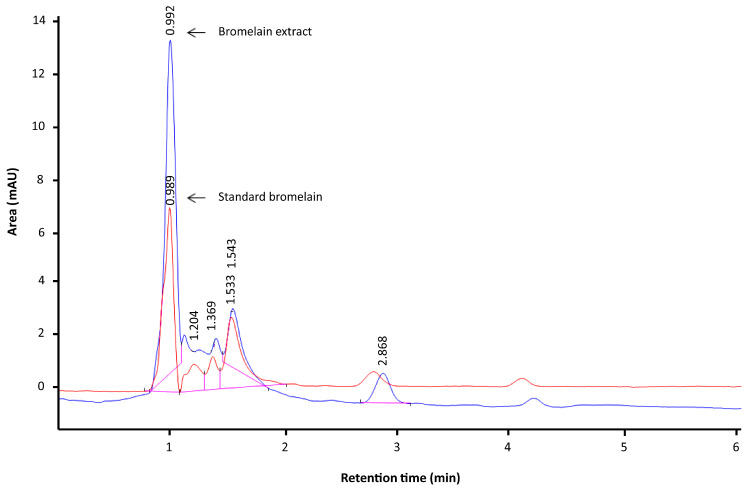
HPLC profile of bromelain extract (blue line) compared with standard bromelain (stem bromelain, red line). The numbers presented in the chromatograms are the retention times of the compounds.

The composition of bromelain varies according to the method of purification and the source. Rhizome bromelain contains higher levels of proteases than bromelain derived from the fruit. The HPLC profile revealed that the bromelain extract used in this study was identical to stem bromelain, and the peak of bromelain extract appeared at a retention time of 0.9 min. The bromelain extract examined in this study was previously characterized for protease activity, and its molecular weight was calculated as 25–27 kDa [10]. The HPLC profile (Figure 1) revealed that bromelain extract was not a pure compound, in line with the results of a previous study reporting that stem bromelain consisted of four proteases, one jacalin-like lectin, and two protease inhibitors according to LC–MS/MS [11]. It is likely that the beneficial effects of bromelain are associated with multiple factors as opposed to a single constituent [12].

### 2.2. Cytotoxicity of Bromelain Extract in Porcine Chondrocytes and Synovial Fibroblasts

**Figure 2:**
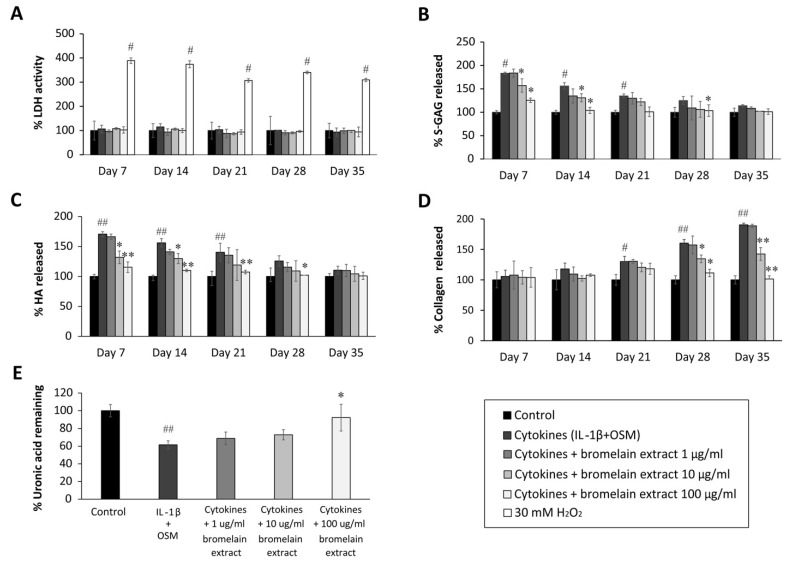
Effect of bromelain extract on the release of glycosaminoglycan release and matrix uronic acid content in porcine explants treated with cytokines (IL-1β/OSM) for 35 days. The culture medium was collected on days 0, 7, 14, 21, 28, and 35 from each group, and cartilage discs on day 35 were digested with 10 U of papain. The LDH activity in culture medium (**A**) was analyzed to determine the toxicity of bromelain compared with H2O2 treatment. The levels of s-GAG (**B**), HA (**C**), collagen released into culture medium (**D**), and uronic acid remaining in cartilage tissue (**E**) were measured and calculated as the percent change versus control. Each value is expressed as the mean ± SD. # *p* < 0.05 compared with control, * *p* < 0.05 compared with IL-1β treatment alone.

Chondrocytes are the residence cells in cartilage, and they participate in OA pathogenesis. The cartilage explants model has been thoroughly used to study the pathology and therapeutic treatment of OA. Chondrocyte lysis mediated by bromelain extract was measured by lactate dehydrogenase (LDH) activity in culture medium. In this study, LDH activity was represented by the LDH levels, and the results illustrated that 35 days of exposure to bromelain extract (1–100 µg/mL) did not significantly increase LDH secretion from chondrocytes in porcine cartilage versus the untreated control. Meanwhile, LDH release was significantly higher following 30 mM H_2_O_2_ treatment (positive control) than in the untreated group (Figure 2A) Thus, bromelain extract (1–100 µg/mL) did not damage chondrocytes in cartilage, making it useful for generating a porcine cartilage explant model.

**Figure 3:**
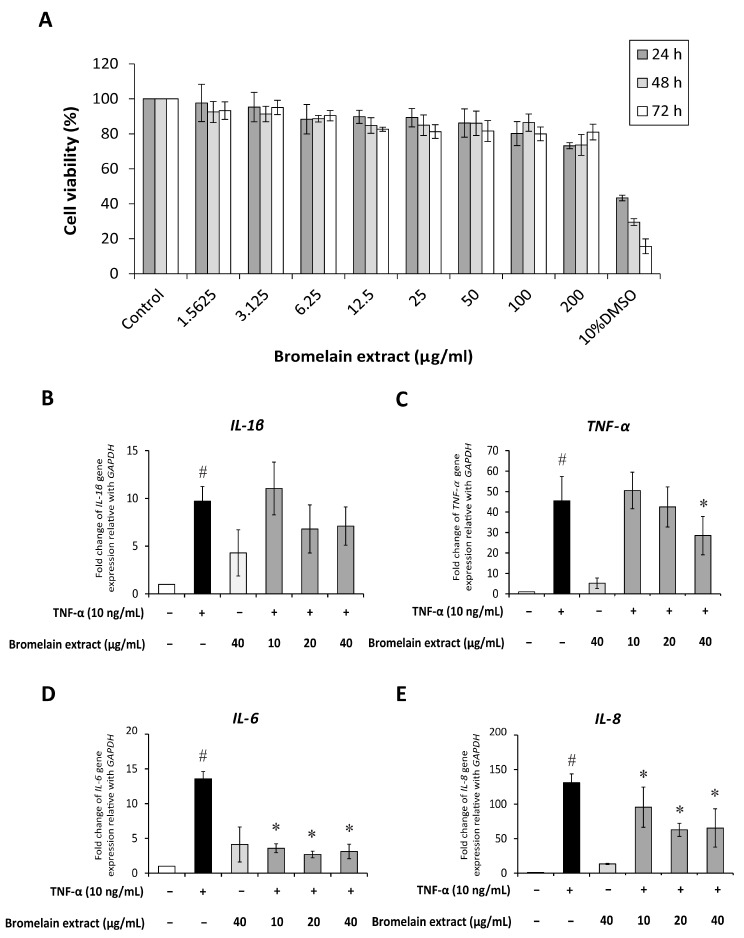
Effect of bromelain extract on cytotoxicity and inflammatory gene expression. The cytotoxicity of bromelain extract in SW982 cells was assessed by the MTT assay (**A**). The effects of bromelain on the gene expression of inflammatory cytokines were investigated by cotreatment with 10 ng/mL human recombinant TNF-α and bromelain extract (10–40 µg/mL) for 4 h. The gene expression of IL-1β (**B**), TNF-α (**C**), IL-6 (**D**), and IL-8 (**E**) was measured by quantitative RT-PCR. Each value is expressed as the mean ± SD. # *p* < 0.05 compared with control, * *p* < 0.05 compared with TNF-α treatment alone.

Synovial fibroblasts participate in RA pathogenesis. Synovial fibroblasts exposed to inflammatory cytokines trigger joint inflammation and cartilage and bone destruction in RA [13]. Human synovial sarcoma cells (SW982) were used to examine the effects of bromelain on TNF-α–mediated inflammation in this study. This cell line is an alternative in vitro model for studying the effects of anti-inflammatory drugs [14,15] and phytochemicals [16] on RA. The 3-(4,5-dimethylthiazol-2-yl)-2,5-diphenyltetrazolium bromide (MTT) assay was assessed to determine the cytotoxicity of bromelain extract in SW982 cells, and the results illustrated that treatment with bromelain extract (1.25–200 µg/mL) for 24–72 h caused no significant cytotoxic effects. The percent cell viability under all conditions relative to the control exceeded 80% (Figure 3A). Thus, this range of bromelain concentrations was used for cell culture experiments.

### 2.3. Chondroprotective Effects of Bromelain Extract

IL-1β induces inflammation in OA, but an in vitro study previously demonstrated that IL-1β, alone, did not induce collagen degradation [17]. In arthritis, oncostatin M (OSM), a cytokine present in synovial fluid and joint tissue, synergizes the action of other inflammatory cytokines (e.g., IL-1, TNF-α, IL-17, lipopolysaccharide (LPS)) [18]. This cytokine participates in inflammatory processes in bone, cartilage, lung, vesicular, and skin diseases [19]. To induce inflammation in this study, porcine cartilage discs were treated with IL-1β/OSM and cotreated with bromelain extract for 35 days. Cartilage explant models are widely used to study the degenerative changes in cartilage tissue [20,21] and therapeutic mechanism [22,23]. In this model, the release of glycosaminoglycans (GAGs) from explants into the cultured medium and the low content of uronic acid and collagen in cartilage explants reflect the induced cartilage degradation.

The sulfated glycosaminoglycan (s-GAG) level in the conditioned medium of IL-1β/OSM-treated cartilage explants was significantly elevated in the early inflammatory stage between days 7 and 14 (Figure 2B) compared with the control level. After cotreatment with bromelain extract (1–100 µg/mL) in the presence of IL-1β/OSM, the results illustrated that high concentrations of bromelain extract (10 and 100 µg/mL) significantly decreased s-GAG release in the cultured medium on days 7–21 compared with the findings in the IL-1β/OSM treatment group. IL-1β/OSM significantly increased the release of hyaluronic acid (HA), the major non–s-GAG, in cultured medium on days 7, 14, and 21 (1.7-, 1.5-, and 1.4-fold, respectively) compared with the control level, and high concentrations of bromelain extract (10–100 µg/mL) significantly inhibited the release of HA on days 7–28 compared with the results in the IL-1β/OSM treatment group (Figure 2C).

The release of degraded collagen in culture medium was significantly higher in the IL-1β/OSM treatment group than in the control group on days 21–35 (Figure 2D). Bromelain cotreatment (10–100 µg/mL) significantly decreased the release of degraded collagen on days 28 and 35 compared with the findings in the IL-1β/OSM treatment group. On day 35, papain-digested cartilage discs exhibited significantly decreased levels of uronic acid (approximately 40%), which influences matrix GAG degradation, in the IL-1β/OSM treatment versus the control level. Upon cotreatment with bromelain extract (100 µg/mL), the uronic acid level in porcine cartilage tissue was significantly higher than that in the IL-1β/OSM treatment group (Figure 2E).

GAGs (s-GAG and HA) and collagen in cartilage matrix are degraded in response to IL-1β/OSM-induced MMP production and activation [24,25,26]. The reduction of IL-1β/OSM-mediated extracellular matrix degradation in the culture medium indicated the chondroprotective property of bromelain extract. In addition, this chondroprotective effect was confirmed by the higher cartilage uronic acid content in the presence of bromelain extract. In clinical trials, oral bromelain was previously described as an analgesic and anti-inflammatory agent for OA and RA [27,28]. Regarding the inflammatory process, the effects of bromelain were reported to involve the reduction of prostaglandin E2 levels [29]. This is the first report to demonstrate the mechanism by which bromelain inhibits cartilage degradation in an ex vivo OA model.

### 2.4. Anti-Inflammatory Effects of Bromelain Extract on TNF-α–Induced SW982

The inflammatory process of the immune system and connective tissue around joint capsules is the crucial mechanism of RA. TNF-α is the principle cytokine that participates in the inflammatory process in synovial tissue [30]. TNF-α, which is produced by immune cells, also plays a pivotal role by inducing inflammation and apoptosis in resident cells in joint capsules [31]. TNF-α is present in high levels in synovial fluid and the pannus in RA [32]. TNF-α over expression in RA has been correlated with synovitis and bone erosion [33] and the production of other inflammatory cytokines [34].

SW982 cells are widely used as an alternative to primary synovial fibroblasts for studying the inflammatory process of RA [16] and examining the effects of anti-inflammatory drugs [14,15]. In this study, the upregulation of inflammatory cytokine (IL-1β, TNF-α, IL-6, IL-8) gene expression was detected in TNF-α–treated SW982 cells (Figure 3C–E). Pretreatment with bromelain extract slightly reduced IL-1β gene expression compared with the findings in cells treated with TNF-α alone. TNF-α gene expression was downregulated by pretreatment with 40 µg/mL bromelain extract. In addition, bromelain extract pretreatment (10–40 µg/mL) significantly downregulated IL-6 and IL-8 gene expression compared with the findings in cells treated with TNF-α alone. Moreover, high-concentration bromelain extract (40 µg/mL) exerted no effects on proinflammatory cytokine expression versus the control.

The effects of bromelain extract on TNF-α in this study agreed with previous findings that bromelain suppressed the expression of IL-1β, IL-6 [6,35], and TNF-α [36] in tissues with induced inflammation. The reduction of cytokine production is one of the therapeutic approaches for RA.

### 2.5. Inhibitory Effects of Bromelain Extract on TNF-α–Induced NF-κB and MAPK Signaling

TNF-α–induced inflammatory responses occur through the NF-κB and MAPK pathways [37]. The production of IL-6 and IL-8 by synovial fibroblasts is a consequence of activation of the NF-κB pathway [38,39]. In addition to the NF-κB pathway, ERK and JNK1 activation contributes to TNF-α–induced IL-8 expression in synovial fibroblasts [40]. Previous studies demonstrated that blockade of TNF stimulation significantly decreased proinflammatory cytokine production in cultured RA synovial cells [41,42,43]. Thus, the effect of bromelain extract on TNF-α–induced NF-κB and MAPK signaling was investigated by Western blotting.

**Figure 4:**
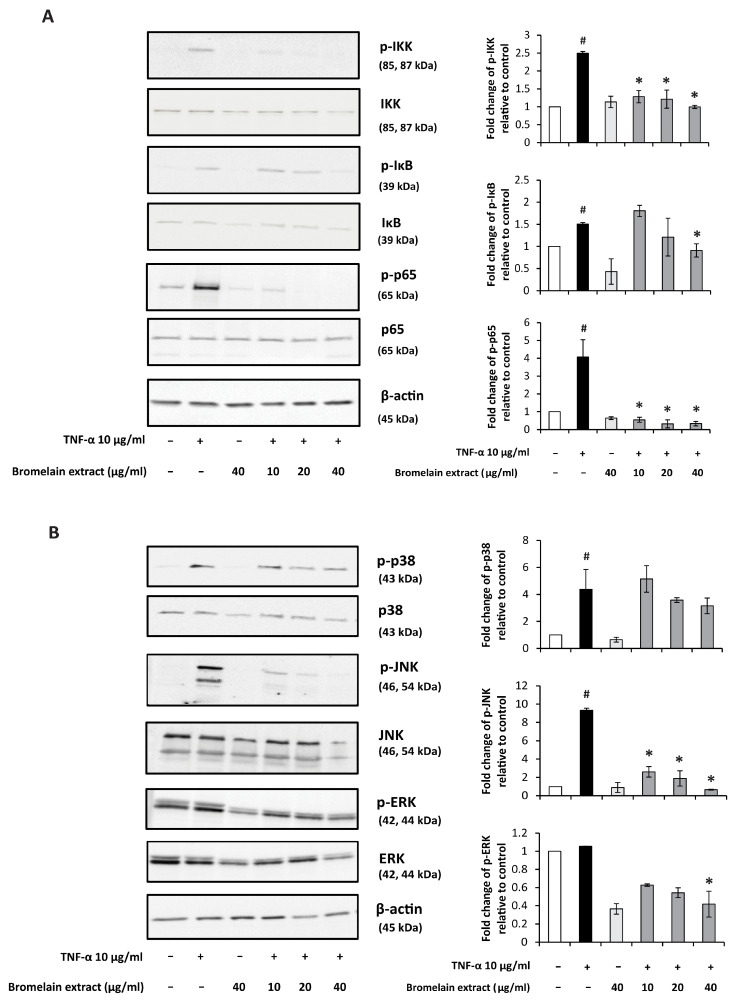
Effect of bromelain extract on the NF-κB and MAPK pathway. The inhibitory effects of bromelain extract 1 h before treatment with 10 ng/mL human recombinant TNF-α for 10 min. The cell lysate was collected and subjected to Western blot analysis(**A**). The density of each band was analyzed using TotalLab TL120 software (**B**). Each value is expressed as the mean ± SD. # *p* < 0.05 compared with control, * *p* < 0.05 compared with TNF-α treatment alone.

In our study, TNF-α treatment activated the NF-κB signaling pathway, and pretreatment with bromelain extract (40 µg/mL) significantly reduced the TNF-α–mediated activation of NF-κB signaling molecules (Figure 4A). Bromelain extract alone had no effects on NF-κB signaling molecule activity. Analysis of the MAPK pathway illustrated that TNF-α activated p38 and JNK signaling in TNF-α–treated SW982 cells. p38 and JNK phosphorylation were reduced when SW982 cells were pretreated with bromelain extract (Figure 4B). Bromelain extract alone had no effect on p38 and JNK phosphorylation. Interestingly, TNF-α exerted no effect on ERK phosphorylation, and treatment with bromelain extract alone decreased ERK activation.

The inhibitory effects of bromelain on NF-κB and MAPK signaling pathways in this study agree with previous findings demonstrating the inhibitory effects of bromelain on the NF-κB pathway and AP-1 molecules [44,45]. Recently, the inhibitory effects of bromelain on LPS-induced NF-κB and MAPK signaling were demonstrated in RAW cells [10,46].

## 3. Discussion

Oral administration of bromelain (12 g/day) can be absorbed with no side effects, and about 40% of the high molecular weight molecules are found in blood circulation [47] Bromelain is absorbed via the human intestines in its intact form with a half-life of approximately 6–9 h [5]. The blood concentration of bromelain peaked 1 h after administration in prior research [48]. The proteolytic activity of bromelain was detected in plasma and was found in the bound form with antiproteinases (2-macroglobulin and alpha1-antichymotrypsin) [49].

In previous research, bromelain extract from pineapple rhizome was characterized and revealed to exhibit protease activity with a molecular weight of approximately 25–27 kDa [10]. In this study, the HPLC profile illustrated that the bromelain extract was identical to commercially available stem bromelain with protease activity and many beneficial biological and pharmacological activities [50].

Regarding joint disease, oral bromelain administration significantly was demonstrated to decrease pain and stiffness in patients with knee OA [51]. The pharmacological mechanism of bromelain was reported as the analgesic influence on bradykinin [52,53], which is a pain-producing substance. In addition, the reduction of pain was an indirect effect of anti-inflammatory actions such as the reduction of edema, debris, and immune responses. A clinical study demonstrated that bromelain produced a significant to complete decrease in soft tissue swelling in patients with arthritic joint swelling [54].

A number of clinical trials also demonstrated that oral administration of bromelain is safe, and it has been used as a food supplement and alternative to NSAIDs in patients with acute inflammation and sports injuries [27]. The antiapoptotic and anti-inflammatory effects of bromelain were demonstrated in primary canine [55] and chondrocyte cell line, SW1353 [56]. However, the molecular mechanism of bromelain in joint resident cells has not yet been reported.

The results of our study supported the anti-inflammatory effects of bromelain on OA and RA. In our OA model, bromelain extract exerted chondroprotective effects against IL-1β by suppressing GAG and collagen degradation. Cartilage extracellular matrix degradation is mediated by MMP overexpression and activation. The degraded molecules are released from cartilage to synovial fluid in addition to the loss of the biological function of cartilage. The suppressive effects of bromelain extract on IL-1β activity and porcine cartilage degradation demonstrated its potential benefits for OA treatment. In an RA model, bromelain extract attenuated TNF-α–induced inflammatory cytokine expression by inhibiting NF-κB and MAPK signaling. The inflammation in synovial fibroblasts is mediated by the TNF-α signaling pathway. The release of proinflammatory cytokines from synovial fibroblasts potentiates the RA pathological process. The blockade of TNF signaling is a clinically verified strategy for treating RA. The mechanism by which bromelain suppresses inflammation in SW982 cells could explain the therapeutic effects of bromelain against RA.

The best biological materials for studying arthritic pathology are human cartilage and primary synovial fibroblasts. Histological analysis of cartilage extracellular matrix molecules and MMPs in IL-1β–treated porcine cartilage tissue should be performed for confirm the chondroprotective effect of bromelain. The protein expression of cytokines released from SW982 cells should be analyzed to verify the anti-inflammatory effects of bromelain. To further investigate the antiarthritic activity of bromelain, an in vivo study using an animal model and investigation of the expression of MMPs and cytokines should be conducted. The findings in this study further support the safety, analgesic activity, and anti-inflammatory effects of bromelain in the treatment of OA and RA.

## 4. Materials and Methods

### 4.1. Chemicals and Reagents

Human recombinant IL-1β and TNF-α were purchased from Preprotech (Rocky Hill, NJ, USA). MTT and stem bromelain were purchased from Sigma–Aldrich (St. Louis, MO, USA). Dulbecco’s modified Eagle’s medium (DMEM), Leibovitz-15 (L-15) medium, fetal bovine serum, streptomycin, and penicillin were obtained from Invitrogen Gibco (Grand Island, NY, USA). Antibodies against p–NF-κB p65, NF-κB p65, p-IκB, IκB, p-ERK, ERK, p-JNK, JNK, p-p38, p38, and β-actin were purchased from Cell Signaling Technology (Beverly, MA, USA) and Thermo Fisher Scientific, Inc. (Rockford, IL, USA).

### 4.2. Preparation of Bromelain Extract from Pineapple Rhizome

Pineapple rhizome was used as raw material and kindly supported from the pineapple factory in Chonburi Province, Thailand. Bromelain enzyme was extracted as previously described [10]. Briefly, pineapple rhizome was chopped into small pieces and mixed with water (1:1 *w*/*v*) to prepare a slurry. After that, the purified bromelain was precipitated by gradually adding ammonium sulfate into the pineapple slurry to achieve 40–80% saturation. The reaction was stirred for 1 h and further incubated at 4 °C overnight. The purified bromelain precipitate was recovered by centrifugation at 4 °C and dried.

### 4.3. Characterization of Bromelain Extracts

Bromelain extract (10 mg) was dissolved in methanol to a volume of 10 mL and then homogenized. The solution was filtered with a 0.45-µm membrane filter and sonicated for 5 min before injection to the HPLC system (Agilent, Santa Clara, CA, USA). Chromatography was performed using a C18 column (100 mm × 4.6 mm), methanol–water (70:30) as the mobile phase with a flow rate of 1 mL/min, and a UV diode array detector at 230 nm [57]. HPLC analysis of bromelain extract was performed in comparison with commercially available stem bromelain.

### 4.4. Cartilage Explants

Articular cartilage from the metacarpophalangeal joints of 3–9-month-old pigs was dissected into 25 cm^3^ discs. After dissection, the cartilage was incubated in DMEM containing penicillin/streptomycin 5% (*v*/*v*) fetal calf serum for 30 min at 37 °C in an atmosphere of 5% CO_2_. The cartilage was incubated in fresh medium for 24 h as a sterile check. Then, cartilage (30–35 mg/well) was cultured in 24-well plates in DMEM for 24 h at 37 °C in an atmosphere of 5% CO_2_.

Cartilage was cotreated with cytokines (10 ng/mL IL-1β and 10 ng/mL OSM) and 1–100 µg/mL bromelain extract. The culture medium was collected and replaced on days 0, 7, 14, 21, 28, and 35 to measure the release of GAGs (sulfated GAG and HA) and degraded collagen (hydroxyproline). Cartilage on day 35 was enzymatically digested with papain (10 units) to analyzing remaining of uronic acid content.

### 4.5. Synovial Sarcoma Cell Line (SW982)

SW982 cells were obtained from ATCC^®^ (number HTB-93). The cells were cultured in L-15 medium supplemented with 10% fetal calf serum at 37 °C in a humidified incubator (NuAire, Plymouth, MN, USA).

### 4.6. Cytotoxicity Assay

The effect of bromelain on chondrocytes was determined by measuring LDH release from cartilage explants into the culture medium. The porcine explants were treated with cytokines (IL-1β + OSM) and bromelain extract (1–100 µg/mL) for 35 days, and 30 mM H_2_O_2_ was used as the positive control. The medium was collected on days 7, 14, 21, 28, and 35, and LDH activity was measured as previous described [58]. Briefly, 625 µL of β-DPNH were mixed with 375 µL of 2 mM pyruvate substrate and incubated at 37 °C for 5 min. After that, 100 µL of culture medium or pyruvate substrate (0–2000 U/mL) were added. After incubation at 37 °C for 30 min, followed by incubation with 1 mL of 2,4-dinitrophenylhydrazine at room temperature for 20 min. Finally, 10 mL of 0.4 N NaOH were added, and samples were incubated at room temperature for 5 min. Hydrazine, which is the product of pyruvate substrate and 2,4-dinitrophenylhydrazine, was measured at an optical density of 445 nm. LDH activity was calculated against a standard curve. The formula for calculating LDH activity is as follows: (LDH activity on each day (day 7, 14, 21, 28, or 35) − LDH activity on day 0)/LDH activity on day 0 × 100.

The cytotoxicity of bromelain extract in SW982 cells was measured using the MTT assay. SW982 cells were plated in 96-well plates at a density of 1 × 10^4^ cells/well. After 24 h, cells were pretreated with various concentrations of purified bromelain for 2 h and subsequently treated with 0–200 µg/mL bromelain extract for 24 h. The cells were incubated with MTT solution at a final concentration of 0.5 mg/mL for 4 h at 37 °C. Then, the supernatant was removed, 100 µL of DMSO were added to dissolve the formazan crystals, and absorbance at 540 nm was determined using a microplate reader (Flow Laboratory, Inc, Rockvile, MD, USA). Untreated cells were used as a control, and viability was set at 100%. The percent cell viability of treated wells was calculated in comparison to the control.

### 4.7. Determination of GAG Release

Sulfated GAG release in the conditioned culture medium from all culture conditions was measured using the dimethyl–methylene blue (DMMB) assay. Briefly, 200 µL of DMMB were added to 50 µL of standard chondroitin sulfate-C (0–40 µg/mL) or culture medium. The complex of DMMB and sulfated GAG was measured using a microplate reader at 520 nm, and the amounts of s-GAG were determined from the standard curve, presented as µg sulfated GAG released/mg cartilage, and correlated with the levels detected in controls. The formula for calculating sulfated GAG release was as follows: sulfated GAG release = (sulfated-GAG level on each day (day 7, 14, 21, 28, or 35) − sulfated GAG level on day 0)/sulfated GAG level on day 0 × 100.

The HA level in cartilage explant culture medium was determined by competitive enzyme-linked immunosorbent assay. By coating the microtiter plates (Maxisorp, Nunc) with 100 µg/mL umbilical cord HA (100 µL/well) in coating buffer at 4 °C overnight. Bovine serum albumin (1% *w*/*v*) in PBS (150 µL/well) was used to block the nonspecific binding area in each well. After washing unbound material, 100 µL of the mixture, either sample (culture medium) or standard competitor (HA Healon^®^: range 19–10,000 ng/mL) in biotinylated hyaluronan-binding proteins (1:200) was added. After incubation for 1 h at room temperature, plates were washed, and then peroxidase–mouse monoclonal antibiotin (100 µL/well; 1:2000) was added and incubated for further 1 h at room temperature. The plates were washed again, and then peroxidase substrate (100 µL/well) was added to allow the color to develop at 37 °C for 5–10 min. The reaction was stopped with 50 µL/well 4 M H_2_SO_4_, and the HA level was calculated as the absorbance ratio at 492/690 nm using the Titertek Multiskan M340 multiplate reader and a standard curve. The percent HA release was calculated using the following formula: HA release = (HA level on each day (day 7, 14, 21, 28, or 35) − HA of day 0)/HA on day 0 × 100.

### 4.8. Measurement of Remaining Uronic Acid Content in Cartilage Explants

The remaining uronic acid content in explants was measured after papain digestion of the cartilage discs using m-hydroxydiphenyl in a colorimetric assay. Specifically, 300 µL of concentrated sulfuric acid–borate reagent were added to standard glucuronic acid lactone (0–40 µg/mL) or the diluted sample, incubated for 15 min at 100 °C, and cooled on ice. Then, 12 µL of carbazole solution were added and incubated for 15 min at 100 °C. The remaining uronic acid content was measured at 540 nm using a microplate reader, and the UA level was determined using a standard curve. The percent uronic acid content remaining was calculated using the following formula: (uronic acid content [g] × dilution factor × dry weight [g])/uronic acid content in control cartilage × 100.

### 4.9. Hydroxyproline Assay

Collagen in condition medium was hydrolyzed with 6 N HCl for 24 h at 100 °C. After hydrolysis, samples were freeze-dried and resolubilized with 50 µL of distilled water. The diluent solution (67% propan-1-ol) and oxidant solution (50 mM chloramine T) was added to the sample. Finally, color reagent (7.5% 4-dimethylaminobenzaldehyde in propan-1-ol) was added to the mixture. The reaction was performed at 70 °C for 10–20 min. The absorbance of 4-dimethylaminobenzaldehyde (the oxidized form of chloramine T) was measured at 540 nm using a microtiter plate reader. The hydroxyproline level was determined from the standard curve. The percent hydroxyproline content was calculated using the following formula: hydroxyproline content = (hydroxyproline on each day (day 7, 14, 21, 28, or 35) − hydroxyproline of day 0)/hydroxyproline on day 0 × 100.

### 4.10. Real-Time Polymerase Chain Reaction (RT-PCR) Assay

SW982 cells were plated into six-well plates until they reached 80% confluence. The culture medium was replaced with serum-free L-15 medium for 24 h. The effects of bromelain on inflammation were investigated by treatment with 10 ng/mL human recombinant TNF-α with or without 10, 20, or 40 µg/mL bromelain extract for 4 h. Total RNA was isolated using RNA isolation reagent (GE Healthcare, Chicago, IL, USA) according to the manufacturer’s instructions. Total RNA (500 ng) of each sample was required for reverse transcription into complementary DNA using iScript™. For RT-PCR, GAPDH was used to normalize the relative expression for determining inflammatory gene expression (Table 1). The PCR protocol consisted of 40 cycles of 5 s at 95 °C, 10 s at 60 °C, and 30 s at 72 °C with an Applied Biosystems 7500/7500 Fast Real-time PCR system using SYBR Greener qPCR Universal.

### 4.11. Western Blotting

SW982 cells were plated into six-well plates until they reached 80% confluence. The culture medium was replaced with serum-free L-15 medium for 24 h prior to pretreatment with serum-free L-15 medium containing 10, 20, or 40 µg/mL bromelain extract for 2 h. Then, human recombinant TNF-α (10 ng/mL) was added to each well, and the cell lysate was collected at 10 min. Cell lysate was harvested using 200 µL ice-cold RIPA buffer containing protease inhibitor and phosphatase inhibitor with scraping, and the protein concentration was determined using the Bradford protein assay. The cell lysate protein (20 µg/well) was loaded into an SDS–PAGE gel (5% stacking gel, 13% separating gel), and electrophoresis was performed. Next, the separated proteins were transferred onto nitrocellulose membranes that were later blocked with 5% (*w*/*v*) nonfat dried milk proteins in PBST. After blocking, various probes, including p65, p-p65, IκB-α, p38, p-p38, JNK, p-JNK, ERK, p-ERK, and β-actin antibodies (Cell Signaling Technology, Inc., Beverly, MA, USA), were added, followed by overnight incubation. The membranes were then washed with PBST, and HRP-conjugated secondary antibodies were added. Incubation was performed for 1 h, and the bands were visualized using enhanced chemiluminescence reagent (GE Healthcare). Quantification of band intensity was performed using TotalLab TL120 software (TotalLab Ltd, Newcastle upon Tyne, UK).

### 4.12. Statistical Analysis

The results were expressed as the mean ± SD from triplicate samples of three independent experiments. Differences between conditions were evaluated by one-way ANOVA. Significance was indicated by *p* < 0.05.

## 5. Conclusions

**Figure 5:**
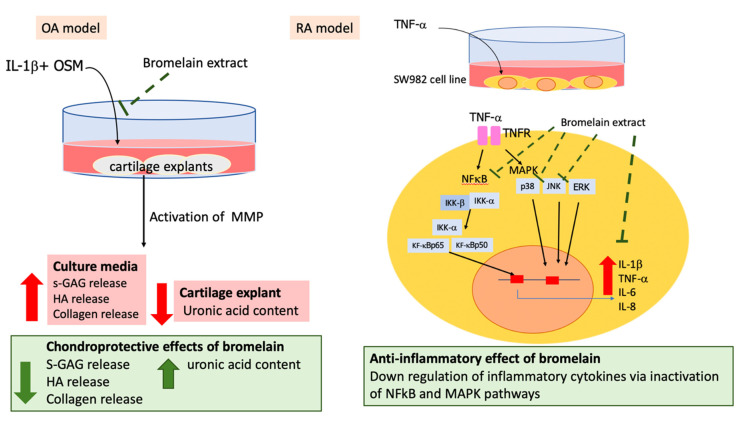
Chondroprotective and anti-inflammatory effects of bromelain extract on IL-1β–treated porcine cartilage explant (OA model) and TNF-α–treated SW982 cells (RA model).

The clinical studies of antiarthritic effects of bromelain were previously demonstrated. In this study, bromelain ameliorated cartilage matrix degradation in inflamed porcine cartilage explant model and attenuated the inflammatory process in synovial fibroblast. The molecular mechanisms of bromelain on cartilage tissue and synovial fibroblast in this study (Figure 5) could explain the chondroprotective and anti-inflammatory effects of oral bromelain in patients with arthritis.

## Figures and Tables

**Table 1:** RT-PCR primers used in this experiment.

Gene	Primer Sequence
GAPDH	F: 5′AGG GCT GCT TTT AAC TCT CGT3′
	R: 5′CCC CAC TTG ATT TTG GAG GGA3′
IL-1β	F: 5′AAA CAG ATG AAG TGC TCC TTC CAG G3′
	R: 5′TGG AGA ACA CCA CTT GTT GCT CCA3′
TNF-α	F: 5′CCC CAG GGA CCT CTC TCT AAT C3′
	R: 5′GGT TTG CTA CAA CAT GGG CTA CA3′
IL-6	F: 5′GGT ACA TCC TCG ACG GCA TCT3′
	R: 5′GTG CCT CTT TGC TGC TTT CAC3′
IL-8	F: 5′CTC TCT TGG CAG CCT TCC3′
	R: 5′CTC AAT CAC TCT CAG TTC TTT G3′

## Data Availability

Data is contained within the article.
